# Abstract of Proceedings of the Buffalo Medical Association

**Published:** 1869-11

**Authors:** T. M. Johnson

**Affiliations:** Sec’y.


BUFFALO

### Medical and Surgical Journal.

VOL. IX. NOVEMBER, 1869. No. 4.

### Original Communications.

ART. I.—*Abstract of Proceedings of the Buffalo Medical Association:*

TUESDAY EVENING, Nov. 2d, 1869.

The President and Vice-President being absent, Dr. JAS. B. SAMO was made Chairman of the meeting.

Members present—Drs. SAMO, LITTLE, GAY, WHITE, ROCHESTER, TAYLOR, BARNES and JOHNSON.

The minutes of last meeting having been published in the JOURNAL, their reading was dispensed with.

Dr. E. R. BARNES read the following paper:

*Gentlemen,*—I present the following report of a case from memory, because it involves some points of interest. It is not an example of good surgery.

In the care of the case, I was associated in a subordinate capacity with a medical gentleman now deceased.

In the month of June, 1866, a boy about fourteen years of age, in stepping from a Brooklyn ferry boat, had his foot caught between the projecting deck of the boat and the dock, or bridge, as it is called.

This bridge has a concave face, corresponding to the convexity of the bow of the boat. In coming in to its dock, the boat generally strikes the bridge a little obliquely, coming into full contact with it only at one point and then more slowly settling into full co-aptation, or, rebounding from the first shock, being drawn into position by chain and windlass.

Had the foot been caught at the point of full contact, it must have been crushed and comminuted by the vast weight of the boat; but from the nature of the injuries sustained, it would seem this was not the case.

Upon examination a few hours after the accident occurred, the foot was found to be swollen and the cellular tissue infiltrated. The foot appeared somewhat shorter than natural, the curvature of the instep was increased, the phalanges were drawn slightly upwards, the metatarsal bones were inclined somewhat inwards. There was a depression over the cuneiform bones, and a laceration through the cellular tissue on the plantar surface of the os-calcis, from which there was a moderate drainage of discolored serum. The temperature of the foot was cool but not cold. It had been immersed in cold water. Reaction soon occurred, and it assumed a natural degree of heat. There was but slight discoloration.

Without going further into details of symptoms, I will state that a subsequent dissection of the amputated limb showed the following conditions. There was an oblique fracture of the first metatarsal bone near its tarsal extremity; the remaining metatarsal bones were fractured obliquely near their phalangeal extremities. There was a dislocation downwards of the three cuneiform bones. The remaining bones of the foot, ankle and joint, and lower part of the leg, were without injury, according to my recollection. There was no comminution of bone.

From the above description, it would seem that the foot was compressed in its long diameter, the toes being turned upwards, and the metatarsal bones borne downwards towards the heel, carrying with them the cuneiform bones. But the foot was not caught at a point of full contact between the boat and deck, or there would have been comminution as well as fracture.

The surgeon in charge determined, unfortunately, as the result proved, to attempt to save the injured member. It was accordingly placed on a pillow, the wound was left open to allow drainage, and the foot was enveloped in cloths wet with tepid water.

On visiting the patient in the evening of the next day, or the day after, (I do not remember which,) the leg was found to be much swollen up to the knee; the skin was tense, and there was a dusky discoloration of the skin extending upward on the inside of the limb from the ankle. The patient complained of a sensation of burning heat at points. Incisions were made through the skin and cellular tissue; but, on the next day, it appeared that the tissues of the leg were extensively involved in rapidly spreading traumatic gangrene, and there were symptoms of much constitutional disturbance. The cellular tissue even above the knee was a little œdematous.

Amputation being now inevitable, the surgeon in charge, in accordance with the advice of Dr. Frank H. Hamilton, performed the operation through the knee joint, leaving the cartilaginous surfaces of the joint intact. The boy quickly rallied after the operation; but an inch and a half of the anterior flap sloughed off, exposing the joint surface to the action of the air. The flaps could not be approximated. A dark spot soon appeared on the face of the outer condyle. The space between the flaps was in time filled up with granulations.

But in the meantime a new complication arose. The patient was attacked with acute pain in the extremity of the stump, occurring at first in paroxysms of brief duration, which came on at nearly the same hour of each day, I was informed. These attacks increased in frequency and intensity, and the stump became swollen and very sensitive to the touch and to motion. The hypodermic injection of morphine controlled the pain, and alone enabled the patient to endure his sufferings. With the lapse of time, the granulating surface showed no disposition to cicatrize, but continued the source of an unhealthy sanious discharge.

Under these circumstances I called upon the surgeon in charge to enquire as to the propriety of re-amputation, but did not receive a favorable reply. A week or two later I called again upon the same errand, but was told that the heat of the weather still rendered delay advisable. I could not coincide with this opinion, and feeling that the patient would be benefitted by such a course, I left the hypodermic syringe with the father, who was familiar with its use, and ceased my visits.

Dr. Louis Bauer subsequently assumed charge and promptly amputated.

The remaining history of this case is taken from the report, in the *Medical Record,* of the New York Pathological Society, at a meeting held January 19th, 1867, Dr. Frank H. Hamilton in the chair. Dr. Bauer presented the specimen under the caption, "Ostitis of lower third of thigh bone, and partial detachment of epiphysis, succeeding amputation at the knee." The patient had suffered eight weeks when he came under his care. He says: The discharge was copious and of a sanious character, the epiphysis (when exposed by the knife) was black, corroded and soft, with but fragments of articular cartilage loosely adherent. The epiphysis, moreover, was evidently loose, and might have been detached without much effort. The subcrurian bursa was slightly distended with fluid. Occasionally the stump would be shaken by spasms of the most painful character. An early amputation of the diseased limb was deemed advisable. Whilst he was under the influence of chloroform, and being prepared for the operation, the stump was still so tender that a gentle touch produced lively reflex tremor. The amputation passed off without any remarkable incident, except there was copious bleeding from the medullary cavity. The periosteum was but loosely adherent, and so much changed in torture, that consecutive troubles of the bone were apprehended. Nevertheless, the wound closed rapidly by first intention, and the patient was about on the fourteenth day.

In reviewing the specimen soon after being removed and longitudinally divided, we found it exhibiting both hyperæmia, intensified by hæmorrhagic depositions, and hyperplasia towards the epiphysis; the articular substance is notably densified, while the periosteum is internally covered with osseous dements—the creation of new bone. On the surface of the bone similar rudimentary attempts are observable. The cartilagincus connection between the shaft and its epiphysis was materially changed in thickness and consistence. Its spontaneous eruption was effected by a maceration of twenty-four hours.

The case may attract more surgical than pathological interest. The injury being limited to the lower part of the leg, left the thigh bone intact. Its diseased condition accrued from the exposure of the epiphysis to the action of atmospheric air and purulent maceration, and thence extended to the shaft. It would seem as if the amputation at the preferred point, under the preceding circumstances, was rather ill conceived, inasmuch as the covering of the stump had to be taken from parts in close proximity to tissues already mortified, and themselves perhaps more or less vitally depressed. Irrespective of this, however, a query arises, whether an amputation at the knee joint before puberty is at all judicious. Dr. Markoe has the merit of having prominently urged this amputation on account of its statistical superiority. The cases adduced in its favor include, if I am correctly informed, two children. The closing of their stumps met with no protraction. I doubt the practicability of so general a rule. From my own experience I infer that there can be no direct union between the flaps and the articular cartilage; the latter has to undergo some prior structural charges, which must protract the cicatrization. While thus suppuration and the formation of granulation tissue is going on, the epiphysis is exposed to the corroding or macerating influence of pus, irrespective of atmospheric air, and may eventually become necrotic. In its turn, secondary amputation would seem to be inevitable. Other surgeons must have had clinical experience to that effect, hence they have suggested abscission of the articular face. I would suggest the revision of the arguments of Dr. Markoe.

Dr. Hamilton asked if the objection of Dr. Bauer to the operation at the knee joint in children, on account of the liability to separation of the epiphysis, would hold good in regard to other joints.

Dr. Bauer stated that he would raise the same objection.

Dr. Sands said that he had operated in two cases on children. Both had done well, notwithstanding that, in one of them, the soft parts used for the flap had been injured and had partly sloughed, while there was besides suppuration in the joint cavity, and exfoliation of the articular lamellæ.

Dr. Bauer was in favor of removing the cartilaginous surfaces of joints in these cases, because: (1) of the liability of the cartilage to become detached, and give rise to dangerous inflammation; and (2) because the whole epiphyseal extremity might become detached, as the results of some morbid process, and death ensue. But the question was an open one.

Dr. Hamilton said that most surgeons held a different opinion, because they thought it less dangerous to leave a cartilaginous surface than to run the risk of inviting an attack of osteo-myelitis by invading the tissue of the bone itself.

Dr. Wood agreed with Dr. Hamilton on this point, but had no experience in amputation at the knee joint.

Dr. Bauer stated that amputation at the knee joint in children had been performed but very few times, and that more experience was needed to fully settle this question.

The discussion terminated at this point.

The above case presents an instance of injury to the foot, followed by traumatic gangrene, and amputation through the knee joint in a lad of fourteen years. There followed disease of the epiphysis and shaft of the thigh bone, with re-amputation and prompt recovery. As exhibiting an interesting series of phenomena, and as eliciting the opinion of a number of eminent surgeons on a question upon which comparatively little has been written, I have thought it worthy of presentation to the society.

Dr. ROCHESTER said that he would take occasion to state, in this connection, that in his opinion there are physicians who use cold applications too generally and too indiscriminately to wounded and inflamed parts. He here related the following illustrative and instructive case:—I was called to Niagara Falls, nine or ten years ago, to see a patient, a lad of sixteen years, who was thrown from the steps of an omnibus into the wheel, producing dislocation at the ————, and severe contusion. I saw him a week after the accident, and found him pale, weak and feeble, and learned that the attending physician had assiduously kept cold application to the limb. Believing, from the condition of the patient, that the cold applications had been injurious, I advised that warm applications be substituted, the good effects of which were soon apparent. The patient made a good recovery. I believe he would have died if the cold applications had been continued for twenty-four hours longer. The application of warm water to sprains is, in my opinion, better practice than the application of cold. Typhoid and typho-malarial fever was reported as prevailing. Dr. ROCHESTER said that he had seen an unusually large number of cases of practice. Had seen six cases within a few days among children from four to ten years of age. Had also seen adults suffering from the same disease. Had treated the first class of cases with doses of one grain of calomel, followed by from four to six grains of citrate of potassa, three times a day.

Had also given anodynes where they were indicated. Had not given quinine, and did not think it generally useful in these cases.

Dr. WHITE said jaundice, in almost every case, is not a disease, but a symptom of a disease. It does not indicate a diminution in the normal amount of bile secreted, but that there is some obstruction to the passage of this fluid into the intestine. I believe that in a majority of these cases, the obstruction is caused by irritation or inflammation of the mucous membrane of the duodenum, at and about the orifice of the ductus choledocus, of sufficient severity to close that orifice and prevent the escape of the bile. Spasmodic closure sometimes also occurs. But if I give cathartics now, they are of the mildest kind. I see no good reason for giving mercury. The alkalies are sufficient. I believe that the less we give active cathartics the better. Counter irritation over the region of the duodenum are beneficial. Give the blandest kind of food. Moderate anodynes are applicable, especially in spasmodic closure. I see no objection to quinine, although it cannot be strictly considered a malarial disease.

Dr. ROCHESTER said that he concurred with Dr. WHITE as to the pathology of jaundice; that it was caused by an obstruction to the free flow of bile into the intestine, and that this obstruction was most frequently produced by inflammation of the mucous membrane of the duodenum. He used calomel not as cholagogue but as a sedative, and he found the relative action of the drug to be induced, sometimes, by small, and sometimes by large doses. He also maintained the fact that the cholagogue action of calomel had been questioned, and that there were some who denied that it had any such action, although, for his part, he did not support this theory.

Dr. BARNES mentioned a case which had continued four weeks, under the use of anodynes, alkalies and external applications, which finally became much aggravated, the pulse being very weak, the food vomited, the bowels constipated, and much pain experienced. Immediate relief to all the symptoms was experienced in this case from a large dose—x x grains of calomel. The patient recovered without the use of further medication. Before commencing the anodyne treatment, laxatives had been used with temporary benefit.

Dr. GAY stated that a committee of European physicians, appointed for the purpose of investigating the effects of preparations of mercury, reported that they do not increase the secretion of bile, but, on the contrary, diminish it.

Dr. ROCHESTER asked if any member had been appointed to prepare a paper on a given subject, to be read before the society. He also said that, when members had accepted such appointment, he thought they should consider themselves bound to read their paper at the meeting designated, and that it would be well for the members generally to read up the subject of the paper, in order that the discussion following the reading might be more instructive and interesting.

Dr. WHITE thought that the Secretary should see that a paper was prepared by some member for each meeting, and that the subject indicated should be written on the notices of the meeting sent to the different members. He moved that Dr. ABBOTT be requested to prepare a paper on some subject in his special department, to be read before the society. Seconded and carried.

Adjourned.

T. M. JOHNSON, *Sec'y.*

